# Supplementary material for: Metformin Inhibited GSDME to Suppress M2 Macrophage Pyroptosis and Maintain M2 Phenotype to Mitigate Cisplatin-Induced Intestinal Inflammation
Source: Biomedicines. 2024 Nov 4;12(11):2526. doi: 10.3390/biomedicines12112526 (PMC11592070; doi:10.3390/biomedicines12112526)
Supplement: Supplementary file 1 [file biomedicines-12-02526-s001.zip › supplemental materials for Biomedicines(1).pdf]

Supplementary Materials for

Metformin inhibited GSDME to suppress M2 macrophage pyroptosis and maintain M2 phenotype to mitigate cisplatin-induced intestinal inflammation

Ke Jiang *et al*.

*Corresponding author. Email: gllsunmo369@sina.com, sqwang@bmi.ac.cn, blg@hbu.edu.cn

This PDF file includes:

Figs. S1 to S4

Tables. S1 to S2

Figure S1

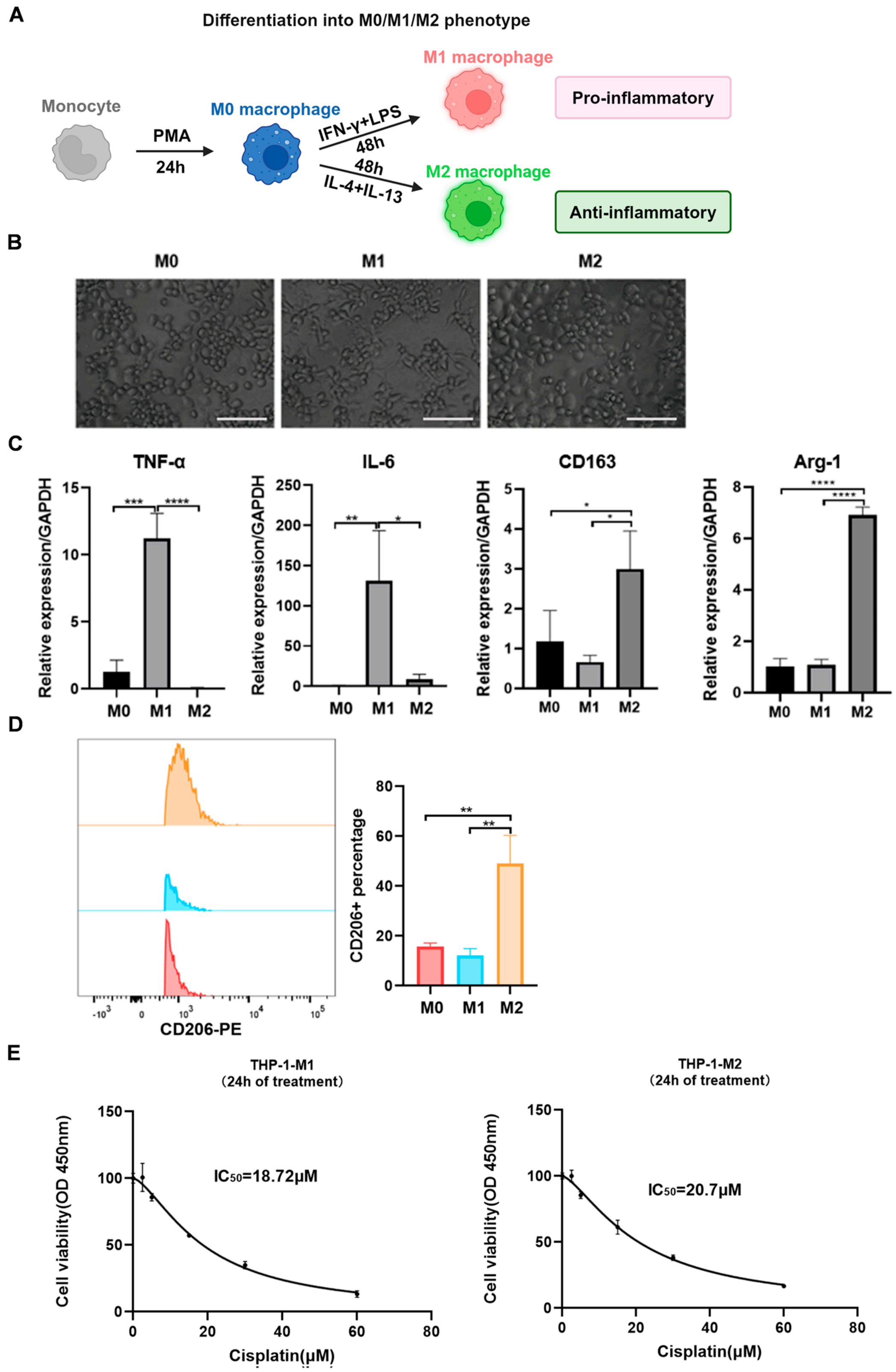
A
Differentiation into M0/M1/M2 phenotype
Monocyte
PMA
24h
M0 macrophage
IFN-γ+LPS
48h
48h
IL-4+IL-13
M1 macrophage
M2 macrophage
Pro-inflammatory
Anti-inflammatory
B
M0
M1
M2
C
TNF-α
IL-6
CD163
Arg-1
Relative expression/GAPDH
M0
M1
M2
D
CD206-PE
CD206+ percentage
M0
M1
M2
E
THP-1-M1
(24h of treatment)
IC50=18.72μM
THP-1-M2
(24h of treatment)
IC50=20.7μM
Cell viability(OD 450nm)
Cisplatin(μM)

Supplemental Figure S1. The polarization of THP-1 cells into different subtypes of macrophages. (**A**) The schematic of the differentiation of the monocytic cell line THP-1 into M0, M1, and M2 macrophages. (**B**) The morphological change of polarized macrophages by the phase contrast microscope. Scale bar: 100 μm. (**C**) The mRNA expression levels of the marker genes for M1 (TNF-α, IL-6) and M2 (CD163, Arg-1) in THP-1-derived M0, M1, and M2 macrophages (*n*=3). (**D**) The flow cytometry analysis of $CD206^+$ population in different subtypes of macrophages (*n*=3). (**E**) The dose–response curve of M1 macrophages and M2 macrophages exposed to cisplatin (*n*=3). P values were calculated by one-way ANOVA with Dunnett's test. $^*P < 0.05$, $^{**}P < 0.01$, $^{***}P<0.001$, $^{****}P < 0.0001$.

Figure S2

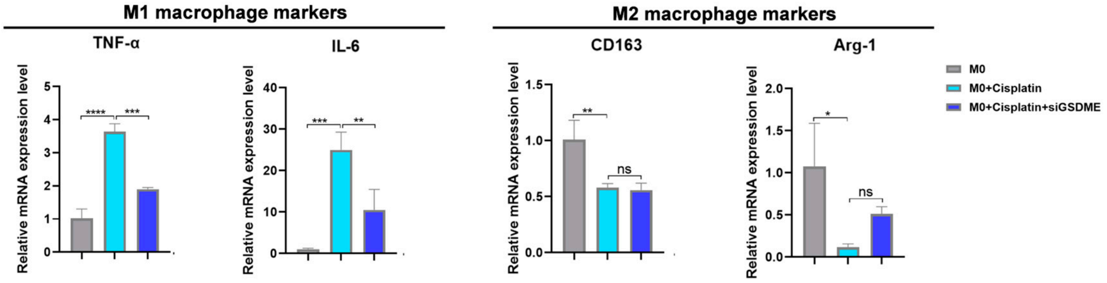

Supplemental Figure S2. The induction of M1- and M2-associated genes in M0 macrophages by cisplatin. The mRNA expression levels of M1 (TNF-α and IL-6) and M2(CD163 and Arg-1) markers were analyzed by qRT-PCR in the control and GSDME-knockdown M0 macrophages upon cisplatin treatment (n=3). P values were calculated by one-way ANOVA with Dunnett's test. *$P < 0.05$, **$P < 0.01$, ***$P < 0.001$, ****$P < 0.0001$, and ns (not significant).

Figure S3

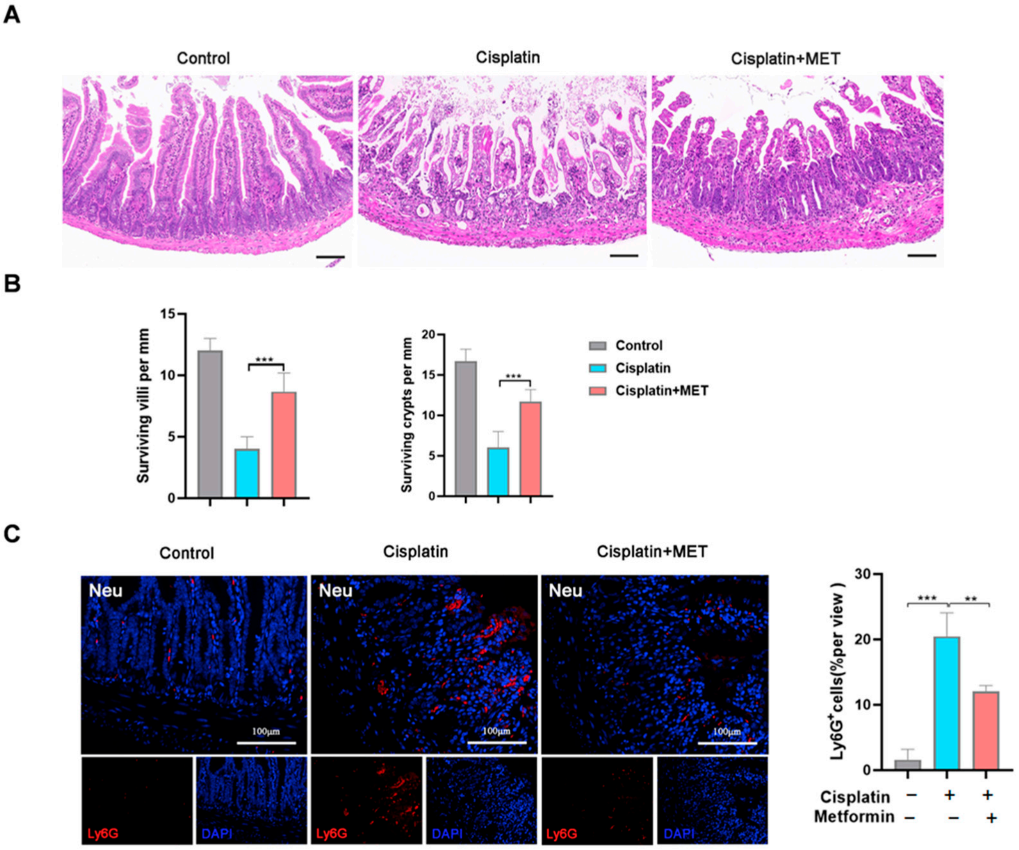

Supplemental Figure S3. The *in vivo* effect of Metformin on cisplatin-induced acute intestinal injury. (**A**) The representative images of the H&E staining of the sections of the small intestine derived from the control, cisplatin-treated, and cisplatin/metformin-treated mice are shown (*n*=5). Scale bar: 100 μm. (**B**) The analysis of the loss of crypts and numbers of surviving villi in different mice models (*n*=5). (**C**) The representative images of immunofluorescence staining of the neutrophils (Ly6g$^+$) in intestinal tissue (*n*=3); scale bar: 100 μm. P values were calculated by one-way ANOVA with Dunnett's test. $^{**}P < 0.01$, $^{***}P < 0.001$.

Figure S4

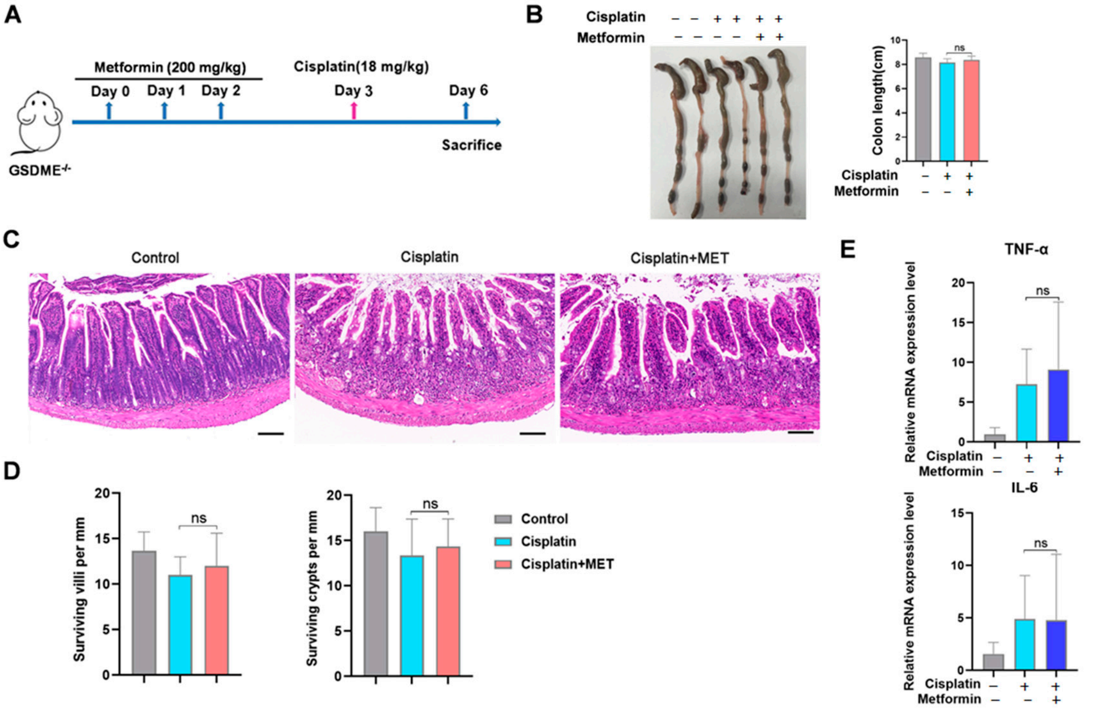

Supplemental Figure S4. Metformin exerted mitigating effects on cisplatin-induced intestinal inflammation through GSDME. (**A**) The schematic of the GSDME$^{-/-}$ mice treated with intraperitoneal injection of metformin and cisplatin at different time points. (**B**) The representative images and the statistical analysis of the colon length of mice from different groups (*n*=6). (**C**) The representative images of H&E staining of the small intestine sections derived from different mice models (*n*=5). Scale bar: 100 μm. **(D**) The analysis of the surviving villi and crypts within the small intestine sections derived from different mice models (*n*=5). (**E**) The mRNA expression of TNF-α, IL-6 in the small intestine of the control, cisplatin-treated, and cisplatin/metformin-treated mice models (*n*=5). P values were calculated by one-way ANOVA with Dunnett's test. ns, not significant.

Supplementary Table S1. Specific primer sequences of qRT-PCR

Gene name	Sequence
TNF-α (human)	Forward: 5'-TCTCGAACCCCGAGTGACAA-3'<br>Reverse: 5'-TGAAGAGGACCTGGGAGTAG-3'
IL-6 (human)	Forward: 5'-CGGGAACGAAAGAGAAGCTCTA-3'<br>Reverse: 5'-CGCTTGTGGAGAAGGAGTTCA-3'
CD163 (human)	Forward: 5'-CCAGAAGGAACTTGTAGCCACAG-3'<br>Reverse: 5'-CAGGCACCAAGCGTTTTGAGCT-3'
Arg-1 (human)	Forward: 5'-GACAGGGCTCCTTTCAGGAC-3'<br>Reverse: 5'-GCCAAGGTTAAAGCCACTGC-3'
GAPDH (human)	Forward: 5'-GCAAATTCCATGGCACCGT-3'<br>Reverse: 5'-GCCCCACTTGATTTTGGAGG-3'
TNF-α (mouse)	Forward: 5'-CGAGTGACAAGCCTGTAGCCC-3'<br>Reverse: 5'-CTCTTTGAGATCCATGCCGTTG-3'
IL-6 (mouse)	Forward: 5'-GCTACCAAACTGGATATAATCAGGA-3'<br>Reverse: 5'-CCAGGTAGCTATGGTACTCCAGAA-3'
GAPDH (mouse)	Forward: 5'-GCAAATTCCATGGCACCGT-3'<br>Reverse: 5'-GCCCCACTTGATTTTGGAGG-3'

Supplementary Table S2. siRNA sequences

Gene name	Sequence
GSDME#1	5’-GCGGTCCTATTTGATGATGAA-3’
GSDME#2	5’-GAUGAUGGAGUAUCUGAUCUUTT-3’
